# NL-VTON: a non-local virtual try-on network with feature preserving of body and clothes

**DOI:** 10.1038/s41598-021-99406-6

**Published:** 2021-10-07

**Authors:** Ze Lin Tan, Jing Bai, Shao Min Zhang, Fei Wei Qin

**Affiliations:** 1 School of Computer Science and Engineering, North Minzu University, Yinchuan, 750021 China; 2 The Key Laboratory of Images & Graphics Intelligent Processing of State Ethnic Affairs Commission, Yinchuan, 750021 China; 3 School of Computer Science, Hangzhou Dianzi University, Hangzhou, 310018 China

**Keywords:** Computer science, Information technology

## Abstract

In an image based virtual try-on network, both features of the target clothes and the input human body should be preserved. However, current techniques failed to solve the problems of blurriness on complex clothes details and artifacts on human body occlusion regions at the same time. To tackle this issue, we propose a non-local virtual try-on network NL-VTON. Considering that convolution is a local operation and limited by its convolution kernel size and rectangular receptive field, which is unsuitable for large size non-rigid transformations of persons and clothes in virtual try-on, we introduce a non-local feature attention module and a grid regularization loss so as to capture detailed features of complex clothes, and design a human body segmentation prediction network to further alleviate the artifacts on occlusion regions. The quantitative and qualitative experiments based on the Zalando dataset demonstrate that our proposed method significantly improves the ability to preserve features of bodies and clothes compared with the state-of-the-art methods.

## Introduction

With the popularity of online clothes shopping, more and more consumers expect to enjoy better shopping experiences by the virtual try-on technique before consumptions. In addition, in the field of fashion design, designers try different clothes on models with different skin tones and body shapes easily through virtual try-on, so as to further inspire or validate their designs. Motivated by these requirements, lots of methods are proposed to solve the virtual try-on problem^1–9^.

One kind of the virtual try-on techniques is based on 3D modeling, which achieved better results based on complete and accurate 3D models. But manual marks and additional hardware devices are required in the process of building 3D models, which leads to limited application prospect.

**Figure 1:**
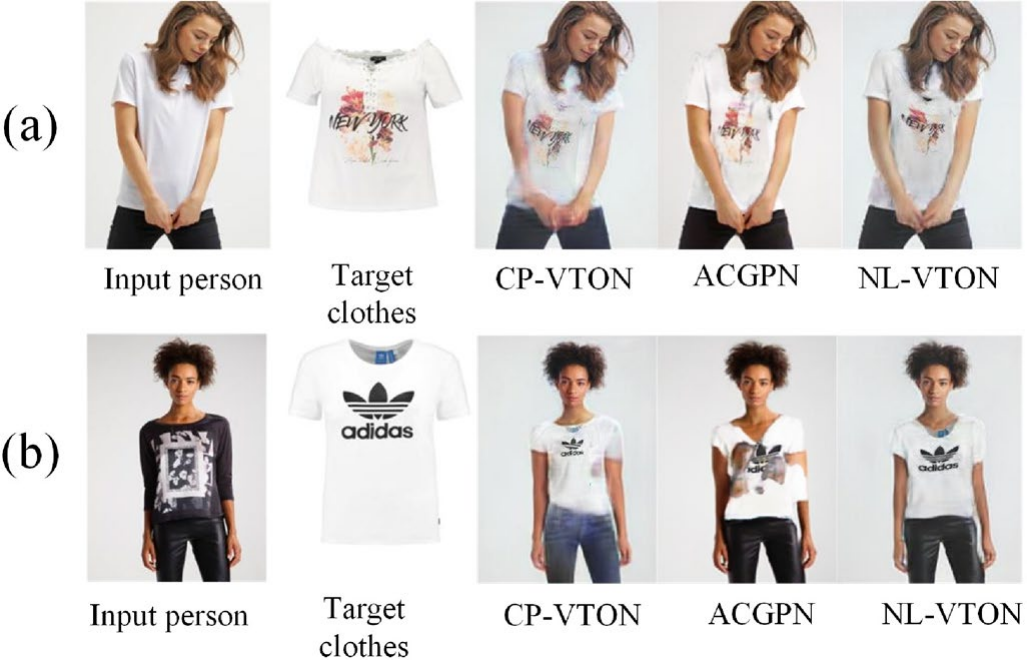
Both in (**a**, **b**) CP-VTON^1^ suffers from a little blurriness on clothes details and serious artifacts on arm regions; ACGPN^9^ alleviates the artifacts on occlusion regions well in (**a**), but cannot deal with long and short sleeves problem and preserves the fine detail features of complex clothes in (**b**); The proposed NL-VTON preserves not only fine details of clothes but also the shapes and posture features of bodies.

Accordingly, another kind of virtual try-on techniques based on 2D images has attracted widespread attentions. They transform the virtual try-on problem into the conditional image generation, with two kinds of items as inputs (one person image and one target clothes image), and generate an image preserving features of the both items. Actually, it is very difficult for 2D virtual try-on networks to preserve different features of the two separate input images at the same time. As shown in Fig. 1a, the classical CP-VTON^1^ network is still affected by a little blurriness on clothes details and serious artifacts on arm regions. Even the advanced method ACGPN^9^, as shown in Fig. 1b which alleviates the artifacts on occlusion regions very well, still cannot preserves the fine detail features of complex clothes.

In all, there is a dilemma between preservation of clothes details and completeness of human bodies, and thus the existing works focus on either details^1–3^ or occlusion problems^4–9^ alone. By analyzing the techniques used in 2D virtual try-on networks, we find there are two issues: (1) They utilize the traditional convolution neural network to generate the final images, in which the convolution is a local operation and limited by its convolution kernel sizes and rectangular receptive fields^10^. Therefore, it is difficult to adapt to large size non-rigid deformations of the input persons and clothes in a try-on problem. (2) Most of the existing works uses GAN (generative adversarial network) to solve the body occlusion problems. It’s tend to preserve arm details so as to keep the occlusion area smooth and thus blures the details on clothes or other interface areas^11^.

In this paper, a Non-Local Virtual Try-on Network (NL-VTON) with feature preserving of both bodies and clothes is proposed based on a human body segmentation prediction module and non-local operations. As shown in Fig. 1, our method not only preserve the global textures and local details on clothes, but also obtains complete body shapes and alleviates the arm occlusion problem simultaneously.

In summary, NL-VTON makes the following contributions:

- A human body segmentation prediction network based on the image inpainting idea is proposed to predict a robust body segmentation of the try-on result, so as to distinguish different regions better and thus capture corresponding features of these regions.
- A novel non-local feature attention module is introduced into different stages of the try-on network, so as to further capture global textures and local detailes of clothes, and obtain complex body posture features.
- A non-local grid regularization loss is designed and applied to the stage of cloth deformation, so as to retain the clothes’ global structeres in the complex clothes deformation process better.
- The quantitative and qualitative experiments based on a standard dataset fully demonstrate that the proposed NL-VTON achieves outstanding performance in feature retention of target clothes and human bodies.

## Related Work

In recent years, with the rapid development of internet economy and artificial intelligence techniques, the fashion analysis and synthesis has shown great potential in practical applications and thus attracts extensive attentions of researchers. Most of the existing studies focus on clothes compatibility and matching learning^12,13^, fashion analysis^14–16^, and virtual try-on^1–6^, among which virtual try-on is the most challenging task.

Previous works on the virtual try-on like Im2fit^17^, are based on 3D and have demonstrated good try-on effects. However, due to their expensive computational costs and recourse requirements^18^, these methods’ application scopes are very narrow. To address the problems, 2D image based virtual try-on methods are proposed, and lots of outstanding works emerge.

The 2D image based methods convert the virtual try-on to a conditional image generation problem. As a pioneer, Jetchev et al.^19^ proposed a conditional analogy GAN and applied it to automatic swapping of clothes on fashion model photos. Over the same period, Han et al. released a new framework VITON^2^ to seamlessly transfer a desired clothes item onto a person using a coarse-to-fine manner. These methods have shown remarkable effect in the virtual try-on problem. However, they still suffers from qualitative defects such as blurriness on clothes details and artifacts on occlusion region of arms.

With the same framework as VITON^2^, CP-VTON^1^ designed a new geometric matching module rather than computing correspondence of interest points as prior works did, which has shown significant improvements in detail preserving of clothes. Wang et al.^3^ illustrated a multi-stage framework to decompose the generation into a spatial alignment and a Tree-Block to harness multi-scale features, which well preserved rich details in salient regions. However, the aforementioned methods have difficulties in handing occlusion problems when the arms cross with the clothes due to the lack of the ability to distinguish body parts.

To distinguish body parts, some works^7,8^ have used a encoder-decoder network to generate human body segmentation, and the state-of-the-art work ACGPN^9^ through the semantic generation module to gradually generate body mask. These works solve the problem of arm occlusion well, but when the target clothes is too different from the model’s clothes, it is difficult to generate accurate segmentation results. We think that this is due to the difficulty of the training and testing phase inconsistency.

Another method, GAN, has also been introduced to solve the the artifacts problem on occlusion regions of arms. Honda et al.^4^ added an adversarial mechanism into VITON in the training pipeline. Raffiee et al.^6^ added two separate GANs including a shape transfer network and an appearance transfer network. Pandey et al.^5^ proposed a new conditional GAN architecture, and Jandial et al.^20^ introduced adversarial loss on texture transfer stage. The work ACGPN^9^ is also based on GAN, which achieves good results by adding semantic generation modules to generate a semantic alignment of spatial layout. These methods demonstrate the superiority of GANs when artifacts arising from improper positioning of the try-on clothes. However, GAN is difficult to achieve Nash equilibrium, and these GAN-based methods still cannot retain fine details of the complex target clothes.

## NL-VTON

**Figure 2:**
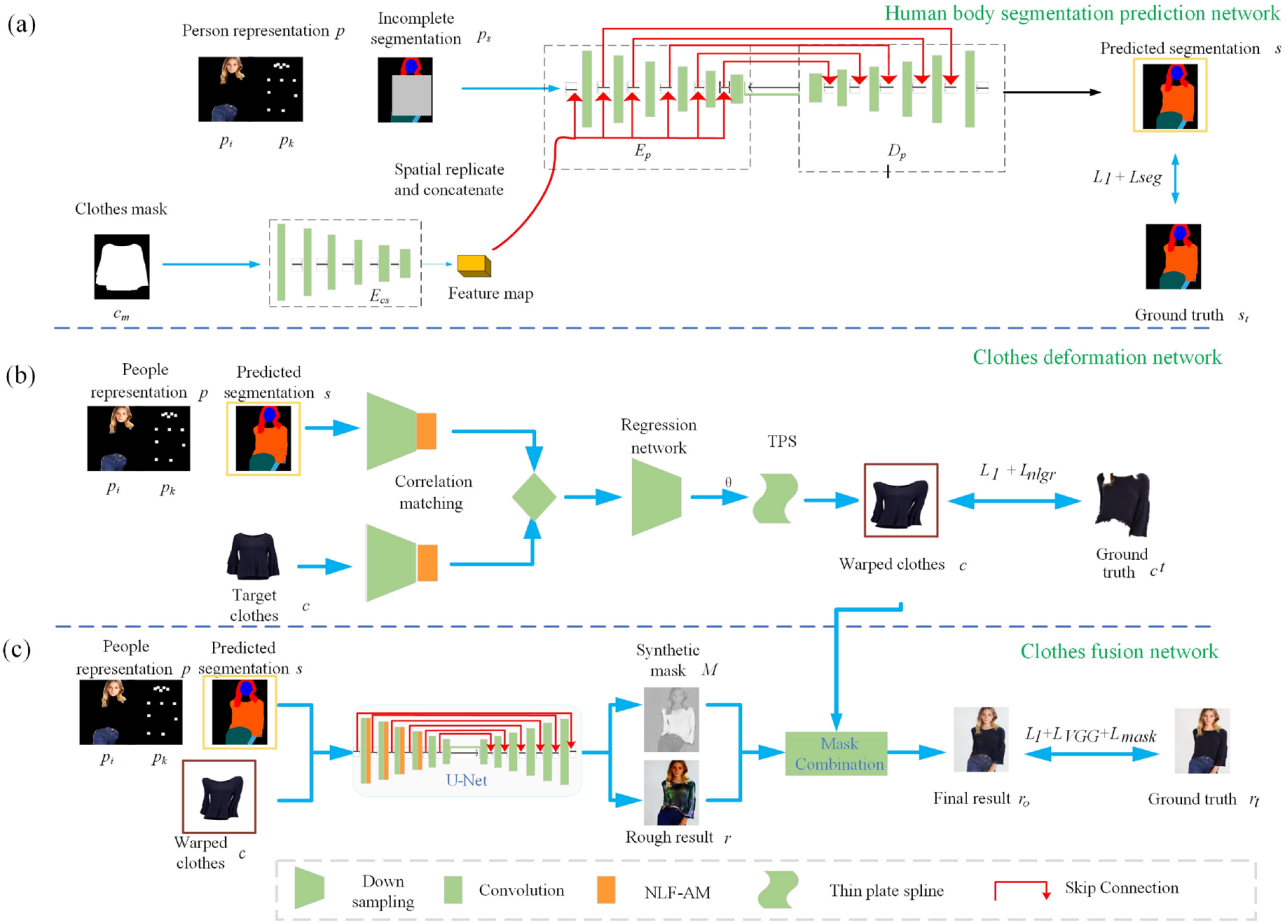
An overview of NL-VTON.

To address the problems of blurriness on clothes details and artifacts on occlusion regions, we propose a Non-Local Virtual Try-On Network (NL-VTON) based on Non-Local Feature Attention Model (NLF-AM), Non-Local Grid Regularization (NL-GR) loss and human body segmentation prediction network. Specifically, as shown in Fig. 2, inputting a person representation, the proposed NL-VTON method consists of three steps in a coarse-to-fine strategie: (1) A human body segmentation prediction network, which predicts the target body segmentation of the try-on result; (2) A clothes deformation network, which generates warped clothes with global texture and local details preservation by introducing a non-local feature attention and a NL-GR Loss; and (3) A clothes fusion network, which generates the final result image with identity of both body shapes and clothes details based on a non-local feature attention and the human segmentation prediction map.

### Person representation

In this paper, in order to effectively retain the overall posture and identity information of the model, the input person representation is composed of four parts as shown in Fig. 2a: (1) The human body posture representation $p_{k}$, which is designed to depict the overall posture of the human body and represented by an 18-channel feature map. Here, each channel is a key point of the human body posture, and corresponds to an 11 * 11 heat map; (2) The identity feature representation $p_{i}$, which depicts the identity information of the persons and corresponds to a RGB image of the head and pants, namely the 3-channel feature map; (3) The human body segmentation representation $p_{s}$, which is the ground truth segmentation with missing specific clothes regions. It is designed to represent the contour of each part of the body and represented by a 20-channel feature map. Here, each channel represents a different part of the body; (4) The target clothes mask representation $c_{m}$, which is designed to represent the contour of the target clothes and represented by a 1-channel feature map.

### Human body segmentation prediction network

**Figure 3:**
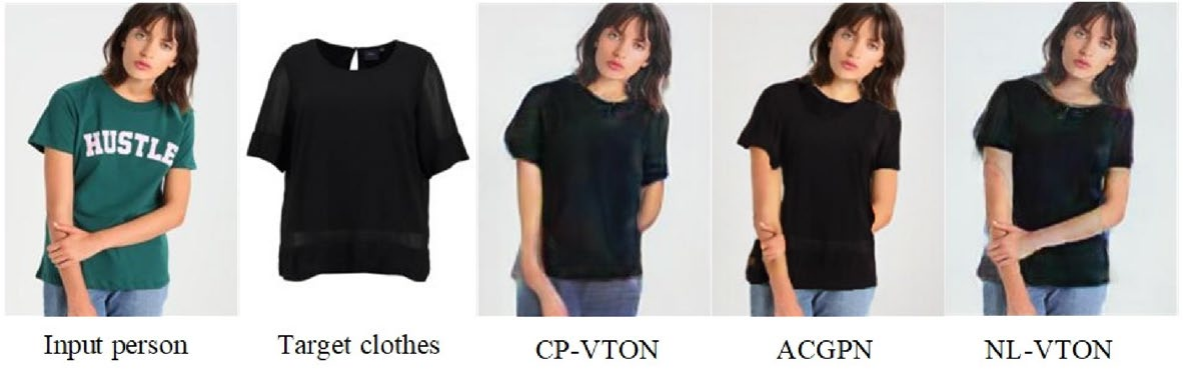
The result of unnatural transformation of other work and NL-VTON. In the try-on result, the arms completely disappear when they cover the clothes.

One of the best methods to solve the arm occlusion in virtual try-on is to generate human body segmentation^7–9^. However, these works are highly depend on the person images with known traget clothes, so it is difficult to generate accurate segmentation results when the target clothes is different from the models’ clothes. An example is shown in Fig. 3, and more visualiztion resutls are shown in experiments.

To solve this problem, we propose a human body segmentation prediction network with a mask to prevent clothes region from attending to segmentation, which ensures that the predicitons for target clothes and arms can depend only on the known other regions. Specifically, network use the person representation of the missing clothes for training and do not include the original clothes information, so the problem is converted into an image inpainting task. It can be used to reasonably predict and deal with artifacts caused by complex poses and occlusion problems. As shown in Fig. 2a, the whole workflow includes the following steps:

- *Step 1.* Inputting the target clothes mask $$c_{m}$$ into the encoder $$ E_{cs}$$ and generating its corresponding feature map $$F_{s}$$, which represents the target clothes contour of the target clothes.
- *Step 2.* Inputting the person representation *p* , including identity feature representation $p_{i}$, body posture representation $p_{k}$ and the incomplete segmentation $p_{s}$, into an encoder-decoder prediction network and output the predicted target segmentation result *s*(a 20-channel human body segmentation map). Here, in order to make the target clothes contour guide the encoding and decoding, we concatenate the feature map $F_{s}$ to each intermediate layer in the encoder after upsamping it by spatially replicating.
- *Step 3.* Calculating the loss between the predicted segmentation result *s* and the ground truth segmentation $$s_{t}$$, and training the network so as to make the output segmentation consistent with the ground truth segmentation. Specifically, the segmentation prediction loss of this stage consists of $$L_{seg}$$ and $$ L_{1}$$, which is formulated as follows:
  1 $$\begin{aligned} Loss_{1}=L_{seg}+L_{1}, \end{aligned}$$
  where $L_{seg}$ represents the cross-entropy loss, which is proposed to make the output and the target image have similar structure, $L_{seg}$ is formulated as follows:
  2 $$\begin{aligned} L_{seg}=\frac{-1}{HW}\sum _{m=1}^{HW}\sum _{c=1}^{C}Slog(S_{t}), \end{aligned}$$
  where *c*, *h* and *w* represent the numbers of channels, height and weight respectively. $L_{1}$ is the sum of distances between pixels on *s* and $s_{t}$, so as to make their details consistent, which is formulated as follows:
  3 $$\begin{aligned} L_{1}=\left| S -S_{t} \right|. \end{aligned}$$

### Clothes deformation network

Having obtained predicted human body segmentation, the clothes deformation network is designed to generate a deformed target clothes that is fitted the input body highly. As mentioned above, existing 2D image based virtual try-on methods heavily rely on the CNN operations, which have rectangular receptive fields and limited kernel sizes. Therefore they are unsuitable for large size non-rigid transformation of the person and clothes in the try-on problem. To solve this problem, we introduce NLF-AM proposed by our group^21^, to break through the spatial limitations of the traditional convolution kernel. It is used to capture the non-rigid features of the human model and realize the feature invariance in the non-rigid transformation of virtual try-on task.

#### The network framework

As Fig. 2b shown, the overall network framework of the clothes deformation network is as follows: with the target clothes *c*, the person representation *p* and the predicted segmentation *s* as inputs, firstly extracting their high-level features through two separately encoders, secondly calculating the correlation between the two high-level features through a correlation matching block and combining them into a single tensor, then predicting the spatial transformation parameter $\theta$ of the thin-plate spline function TPS (Thin-Plate Spline) through a regression network, finally obtaining the warped clothes $c'$ by the TPS transformation using the learned parameter $\theta$.

Compared with the CP-VTON^1^, this paper makes improvements in the following three aspects: (1) Inputting human body predicted segmentation maps rather than a simple body shape mask; (2) Addings NLF-AM into the person encoder and the clothes encoder, respectively, so as to further capture the global attributes and local salient features of human bodys and clothes; and (3) Adding a new NL-GR loss function for clothes fidelity based on the $L_{1}$ loss to make the warped clothes have a global texture identity with the target clothes.

#### Non-local feature attention module

**Figure 4:**
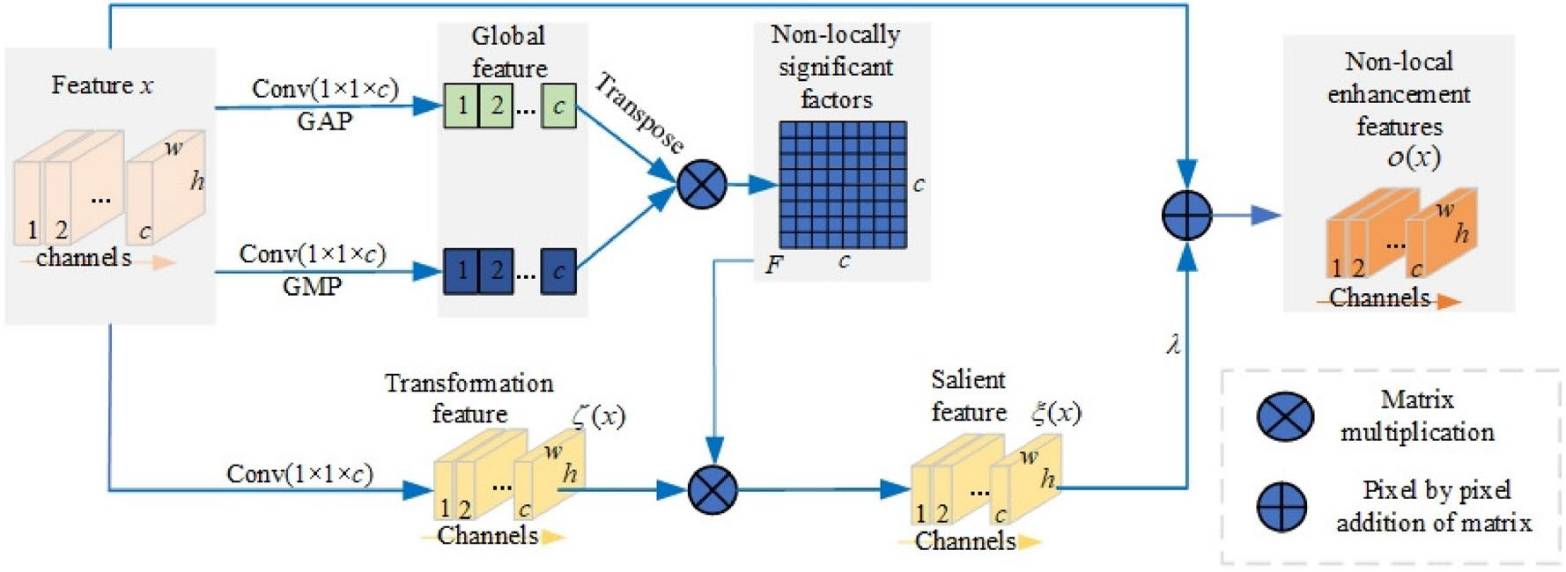
The non-local feature attention module (NLF-AM).

As shown in Fig. 4, inputting a feature map *x*, NLF-AM constructs its non-local enhancement feature *o*(*x*) through the following three steps:

*Step 1.* Constructing the non-local significant factor matrix F. For any input *x*, we firstly generate two embedding features by a 1 * 1 convolution with a global average pooling and a 1 * 1 convolution with a global max pooling, respectively, and then the two feature maps are transposed and multiplied to obtain the correlation matrix F between different features.

The $i_{th}$ row in the matrix reflects the correlation between the features of channel *i* and the features of other channels. Here, the significant feature is further strengthened through multiplication operation. In addition, the matrix F has the excellent characteristic of “breaking through the limitation of convolution kernel size and rectangular receptive field, and capturing the long-distance dependence between features”, named as non-local significant factor matrix in this paper.

*Step 2.* Calculating the salient feature $\delta$(x) by weighting the transformed feature maps $\delta$(x) with the non-local significant factor matrix F. Since F breaks through the limitation of the local receptive fields and captures the global dependence relationship between features, the above weighting operation can further capture the response of each point to the global salient feature.

*Step 3.* Constructing the non-local enhancement feature *o*(*x*) by a weighted residual connection, formulated as $o(x)=\lambda \xi (x)+x, \lambda \in [0,1]$. Obviously, the output feature not only retains the local feature information of the original input features, but also reflects the non-local salient features to a certain extent, that is, the global attribute features.

#### Clothes deformation loss

In the training phase, the pixel-level $L_{1}$ loss is used to evaluate the consistency between the warped clothes $c'$ and the ground truth clothes $c_{t}$, which is formulated as follows:

4 $$\begin{aligned} L_{clothes}(\theta )=\left\| c^{'}-c^{t} \right\| _{1}=\left\| TPS(c,\theta )-c^{t} \right\| _{1}, \end{aligned}$$

where $\theta$ represents the TPS transformation parameter learned by the network. This loss requires that the corresponding pixels between the warped clothes and the ground truth clothes are as close as possible. However, when the target clothes contain complex details or striped patterns, it may result in local deformation. Analyzing deeply, we can find that is because the above $L_{1}$ loss only considers the consistency of pixel levels but ignores the consistency of local structures, which may cause deformation of local shape features or global texture features.

**Figure 5:**
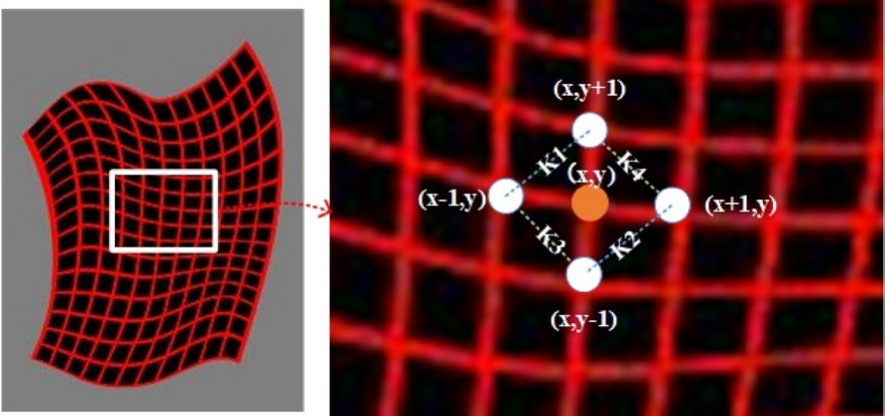
Ilustration of the NL-GR loss function. The figure above shows the distance and slope between the four adjacent points of the constraint grid point (x,y).

In order to eliminate above problem, we add a new NL-GR loss function $L_{nlgr}$ for clothes fidelity. As shown in Fig. 5, this loss function acts on the TPS deformed grid. The minimal of this loss pushes the horizontal, at the same time, vertical Laplace regularization terms and the second order difference terms (k1–k2) and (k3–k4) be as small as possible, which is formulated as follows:

5 $$\begin{aligned} \begin{aligned} L_{nlgr}(\theta )&=\lambda _{a}(\left| |D (x+1,y)-D(x,y) \right| -\left| D (x,y)-D(x-1,y) \right| |\\&\quad + \left| |D (x,y+1)-D(x,y) \right| -\left| D (x,y)-D(x,y-1) \right| |)\\&\quad + \lambda _{b}(\left| K1-K2 \right| +\left| K3-K4 \right| ), \end{aligned} \end{aligned}$$

where D(x,y) represents the values of coordinate point in the grid; $\lambda _{a}$ and $\lambda _{b}$ represent the weighting factors, which are used to adjust the ratio between Laplace regular terms and the second order difference terms.

Based on the above analysis, the final clothes deformation loss in this stage can be formulated as follows:

6 $$\begin{aligned} Loss_{2}=\lambda _{c}L_{clothes}(\theta )+\lambda _{n}\lambda _{nlgr}(\theta ), \end{aligned}$$

where $\lambda _{c}$ and $\lambda _{n}$ represent weighting factors, which are used to adjust the ratio of pixel-level loss and non-local clothes fidelity loss.

**Figure 6:**
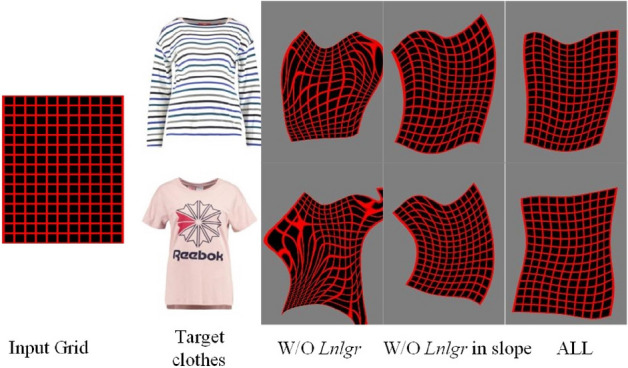
Clothes deformation network comparison results before and after adding the NL-GR loss.

Figure 6 shows the comparison results before and after adding the proposed NL-GR loss $L_{nlgr}$. The 1st column is the input grid, the 2nd column shows two target clothes images; the 3rd column shows the outputted deformed grid without the NL-GR loss, the 4th column shows the outputted deformed grid only using the Laplace regularization terms, and the last column shows the outputted deformed grid using complete NL-GR loss $L_{nlgr}$. Compared the results in three different cases, it can be found that after adding Laplacian regularization terms in the horizontal and vertical directions, the results of the deformed meshes are more regular, and their symmetry in local areas can be further improved by increasing the second order difference terms.

**Figure 7:**
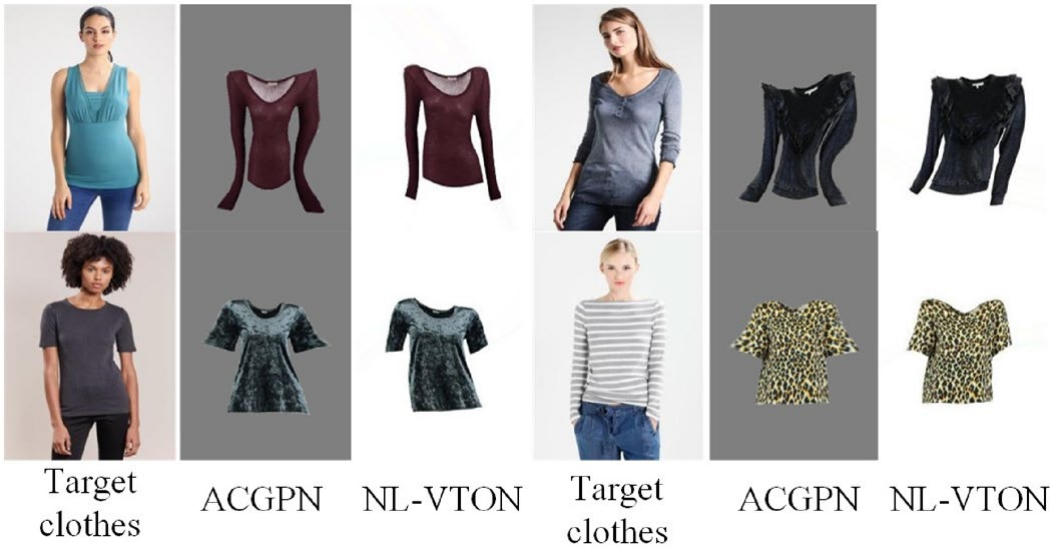
Clothes deformation results by ACGPN^9^ will creates unnatural deformation so it cannot preserve structures of the target clothes better.

ACGPN^9^ also proposes a second-order difference loss acting on the grid, we all constrain the distance between the center point with two adjacent points on each axis and make the grid deformation more regular, but in terms of slope, the loss in ACGPN minimizes the slope difference between the two lines diverging from the center point of each axis, so it will make the shape formed by the center point be constrained to be a rectangle. For NL-GR loss, We respectively constrain the disjoint lines adjacent to the center point to be as parallel as possible, so it will make the shape formed by the center point be constrained to be a parallelogram. From the above analysis, it can be seen that the loss in ACGPN is more restrictive, and it is difficult to adapt to the complex situation in the clothes deformation process. This can be seen in Fig. 7.

### Clothes fusion network

The warped clothes $c'$ obtained from the clothes deformation network roughly fits the body shape of the input person. In this section, we will further integrate the human body feature and generate more realistic results by the clothes fusion network.

Figure 2c shows the network structure, which consists of the following two coarse-to-fine steps:

- *Step 1.* Constructing a rough try-on result *r* and a synthesis Mask *M* through a U-Net^22^ encoder-decoder network with the inputs of person representation *p* (same as clothes deformation network), warped clothes $c'$ and the predicted segmentation *s*. Here, M is the last layer of the output feature maps.
- *Step 2.* Generating the final try-on result $r_{o}$ by fusing the rough try-on result $r_{o}$ and warped clothes $c'$ using synthetic mask M, which is formulated as follows:
  7 $$\begin{aligned} r_{o}=M\otimes c^{'}+(1-M)\otimes r, \end{aligned}$$
  where $\otimes$ represents the multiplication operation between corresponding elements in the two matrices.

It should be noted that in order to fully capture the key features of the human body and the target clothes, different from the traditional encoder-decoder network, we add the NLF-AM into the first four layers of the encoder in a U-Net network to focus the salient features and improve the quality of the final composite image better.

In the training phase, in order to minimize the difference between the generated result $r_{o}$ and the ground truth image $r_{t}$, the same as CP-VTON^1^, the loss function includes three parts: the $L_{1}$ loss at the image pixel level, the VGG perception loss at the semantic level, and a $L_{1}$ regularization loss for mask *M*.

The image pixel level $L_{1}$ loss is defined as follows:

8 $$\begin{aligned} L_{1}(r_{o},r_{t})=\left| r_{o}-r_{t}\right| , \end{aligned}$$

The VGG perceptual loss^23^ describes the semantic difference between two images by calculating the distance between features extracted by a VGG network. In this paper, we make a coarse-to-fine feature constraint by adding a VGG loss between the rough try-on result *r* and ground truth $r_{t}$ besides the VGG loss between final try-on result $r_{o}$ and the ground truth image $r_{t}$, which are formulated as follows:

9 $$\begin{aligned} L_{VGG}(r -r_{t})= & {} \sum _{i=1}^{5}\lambda _{1}\left\| \delta _{i}(r-\delta _{i}(r_{t})) \right\| _{1}, \end{aligned}$$

10 $$\begin{aligned} L_{VGG}(r_{o}-r_{t})= & {} \sum _{i=1}^{5}\lambda _{1}\left\| \delta _{i}(r_{o}-\delta _{i}(r_{t})) \right\| _{1}, \end{aligned}$$

where $\delta _{i}(r)$ represents the *i*th-layer feature map in the visual perception network VGG19^24^ (a network model pre-trained by ImageNet). The value of *i* from 1 to 5 represent ’$conv1_2$’, ’$conv2_2$’, ’$conv3_2$’, ’$conv4_2$’ and ’$conv5_2$’, respectively.

Combining both low-level and high-level image features in VGG loss with image pixel level loss, both attributes, local structures and global contents between the generated image and the ground truth image are considered in this stage. Furthermore, in order to retain the feature of the target clothes as much as possible, $L_{1}$ regularization loss on the mask *M* is added. Finally, the overall loss of the clothes fusion subnetwork in this stage are formulated as follows:

11 $$\begin{aligned} \begin{aligned} Loss_{3}&=\lambda _{L_{1}}\left\| r_{o}-r_{t} \right\| _{1}+\lambda _{VGG}((r_{o},r_{t})+(r_{},r_{t}))\\&\quad +\lambda _{mask}(\left\| 1-M \right\| _{1}, \end{aligned} \end{aligned}$$

where $\lambda _{L_{1}}$, $\lambda _{VGG}$ and $\lambda _{mask}$ are weighting factors, which are used to adjust the ratio of ${L_1}$ loss, ${L_{VGG}}$ loss and ${L_{mask}}$ loss.

## Experiment and evaluation

### Experiment setup and evaluation metrics

#### Dataset

All experiments in this paper are based on the Zalando dataset proposed by Han et al.^2^. The dataset contains 16,253 frontal-view woman and top clothes image pairs, of which 14,221 pairs are used for training and 2032 pairs are used for testing. The resolution of each image in the dataset is 256 * 192. In the virtual try-on datasets, there is no paired data, which means that there is no model wears two or more different clothes. Therefore, in the qualitative comparison, when the target clothes is different from the original model’s clothes, there is no ground truth; while in the quantitative comparison, the target clothes is chosen the same as the original model’s clothes, and the ground truths used to calculated the metrics are original pictures.

#### Training setup

In all experiments, we set batch size to 8 and use ADAM optimizer with $\beta _{1}=0.5$, $\beta _{2}=0.999$ for the three networks, and set the maximum number of iteration steps to 2*$10^{4}$ epochs and the learning rate to 0.0001 for segmentation prediction network. For the other two networks, we set the maximum number of iteration steps to 2*$10^{5}$ epochs, the initial learning rate to 0.0001 and uniform linearly decrease it to 0 after 1*$10^{5}$ epochs. In formula 1, the weighting factors are not specifically indicated, so they are all 1; In formula 5, $\lambda _{a}$ is 1, $\lambda _{b}$ is 1/3; In formula 6, $\lambda _{c}$ is 1, $\lambda _{n}$is 40; In Eq. (11), $\lambda _{L_{1}}$, $\lambda _{VGG}$ and $\lambda _{mask}$ are all 1.

The specific structures of the three networks are shown in Tables 1, 2 and 3. It is noted that in order to reduce the checkerboard effect, we implement the upsampling operations of decoder in the clothes fusion network using the combination of nearest neighbor interpolation and convolution with step size of 1 rather than traditional deconvolution.

**Table 1:** The structure of semgementation prediction network.

Input	Clothes mask 1 * 192 * 256 for the encoder $E_{cs}$	Person representation 39 * 192 * 256 for the encoder $E_{p}$
Encoder ($E_{cs}$,$E_{p}$has same structure)	ResBlock start 32 * 64	
	ResBlock down 64 * 128	
	ResBlock down 128 * 128	
	ResBlock down 128 * 128	
	ResBlock down 128 * 128	
	ResBlock down 128 * 40	
	Conv1 * 1–20 (s = 1)	
Decoder	ResBlock up 128 * 128	
	ResBlock up 128 * 128	
	ResBlock up 128 * 128	
	ResBlock up 128 * 64	
	ResBlock up 63 * 32	
	Conv3 * 3–20 (s = 1)

**Table 2:** The structure of clothes deformation network.

Input	Person representation 41 * 192 * 25	Target clothes 3 * 192 * 256
Feature extraction network	Conn 4 * 4-64 (s = 2)	
	Conv 4 * 4-64 (s = 2) + BN+ RELU	
	Conv 4 * 4-128 (s = 2) + BN + RELU	
	Conv 4 * 4-512 (s = 2) + BN + RELU	
	Conv 3 * 3-512 (s = 1) + RELU + BN	
	Conv 3 * 3-512(s = 1) + RELU+FA	
Regression network	Conv 4 * 4-512 (s = 2) + BN + RELU	
	Conv 4 * 4-256 (s = 2) + BN + RELU	
	Conv 3 * 3-128 (s = 1) + BN + RELU	
	Conv 3 * 3-64 (s = 1) + BN + RELU	
	FC:output-50 + Tanh

**Table 3:** The structure of clothes fusion network.

Input	Person representation 41 * 192 * 25	Target clothes 3 * 192 * 256
Down sampling	Conn 4 * 4-64 (s = 2) + FA	
	Conv4 * 4-128(s = 2) + LeakyReLU + BN + FA	
	Conv4 * 4-256(s = 2) + LeakyReLU + BN + FA	
	Conv4 * 4-512(s = 2) + LeakyReLU + BN + FA	
	Conv4 * 4-512(s = 2) + LeakyReLU + BN	
	Conv4 * 4-512(s = 2) + LeakyReLU	
Up sampling	Conv3 * 3-512(s = 1) + RELU + Bilinear + IN	
	Conv3 * 3-512(s = 1) + RELU + Bilinear + IN + Dropout(0.5)	
	Conv3 * 3-256(s = 1) + RELU + Bilinear + IN + Dropout(0.5)	
	Conv3 * 3-128(s = 1) + RELU + Bilinear + IN	
	Conv3 * 3-64(s = 1) + RELU + Bilinear + IN	
	Conv3 * 3-4(s = 1) + RELU + Bilinear + IN	

#### Evaluation metrics

In this paper, we use two metrics and one visualization methods widely used in virtual try-on to quantitatively and qualitatively compare the effects of the proposed method in this paper.

- SSIM^25^: structural similarity index, the luminance, calculates the contrast and structural similarities between the generated image and the real image to comprehensively evaluate the similarity between the two images. The higher the SSIM index is, the better the image is generated.
- FID^26^: Fréchet Inception Distance, calculates the features’ distance between the generated image and the real image generated by a pre-trained Inception-V3 network. The lower the FID, the smaller the distance between the generated image and the real image, which means the higher quality and the better diversity of the generated image.

### Comparison with the state-of-the-art methods

In order to validate the proposed method, we select CP-VTON^1^, GarmentGAN^6^ and ACGPN^9^ as our compared methods in view of their state-of-the-art performances. All the experiment results of CP-VTON and ACGPN are obtained by downloading and running their source codes; and GarmentGAN does not provide the source code, so its quantitative and visualization results are from its paper.

#### Quantitative results

The quantitative comparative results with the state-of-the-art methods are shown in Table 4. The SSIM for GarmentGAN^6^ is empty because their authors do not provide the corresponding values in their paper. For FID, NL-VTON has the best value, it achieves 8.922 improvement to CP-VTON and 2.415 improvement to GarmentGAN. For SSIM, NL-VTON achieves 0.047 improvement to CP-VTON. And compared with ACGPN, the value is very close to FID and SSIM, so more results are presented in qualitative comparison. From Table 4, we can observe that the validity of the proposed NL-VTON in the virtual try-on.

For the model complexities, compared to the baseline model CP-VTON, all the performance gains of NL-VTON are only based on 12M parameters costs, which is less than 4.4$\%$ of the parameter numbers containing in original network. Furthermore, compared to the advanced network ACGPN consisting of 3 encoder–decoder networks, 3 discriminators and a spatial transformer network, our network only consists of 2 encoder–decoder networks and a clothes deformation network, which is less complex than ACGPN obviously.

**Table 4:** The quantitative evaluation of virtual try-on.

Method	FID	SSIM	FLOPS
CP-VTON	23.085	0.796	279M
GarmentGAN	16.578	N/A	N/A
ACGPN	14.155	0.845	N/A
NL-VTON	14.163	0.843	291M

#### Qualitative results

**Figure 8:**
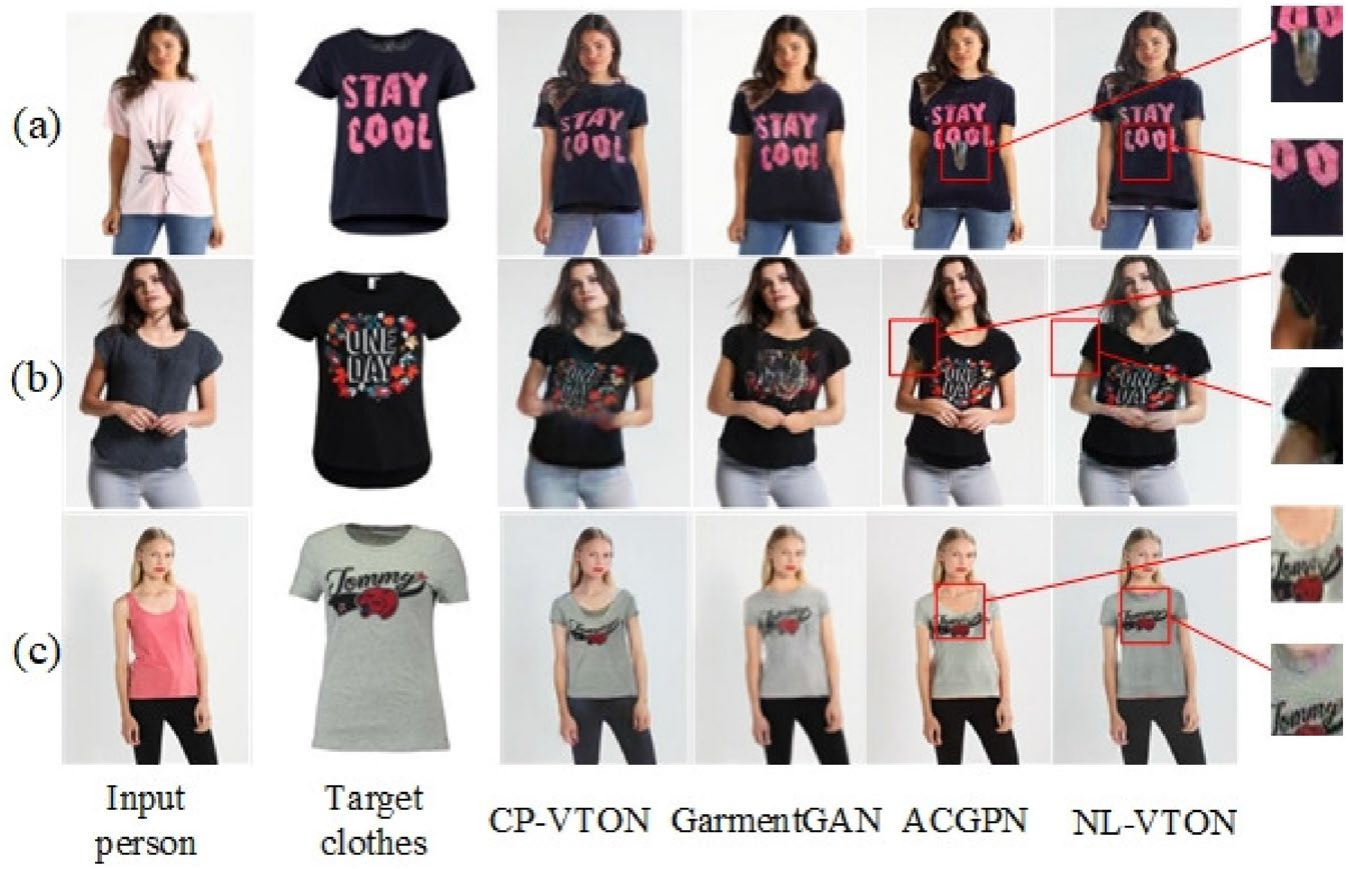
The visual comparison of four different methods.

**Figure 9:**
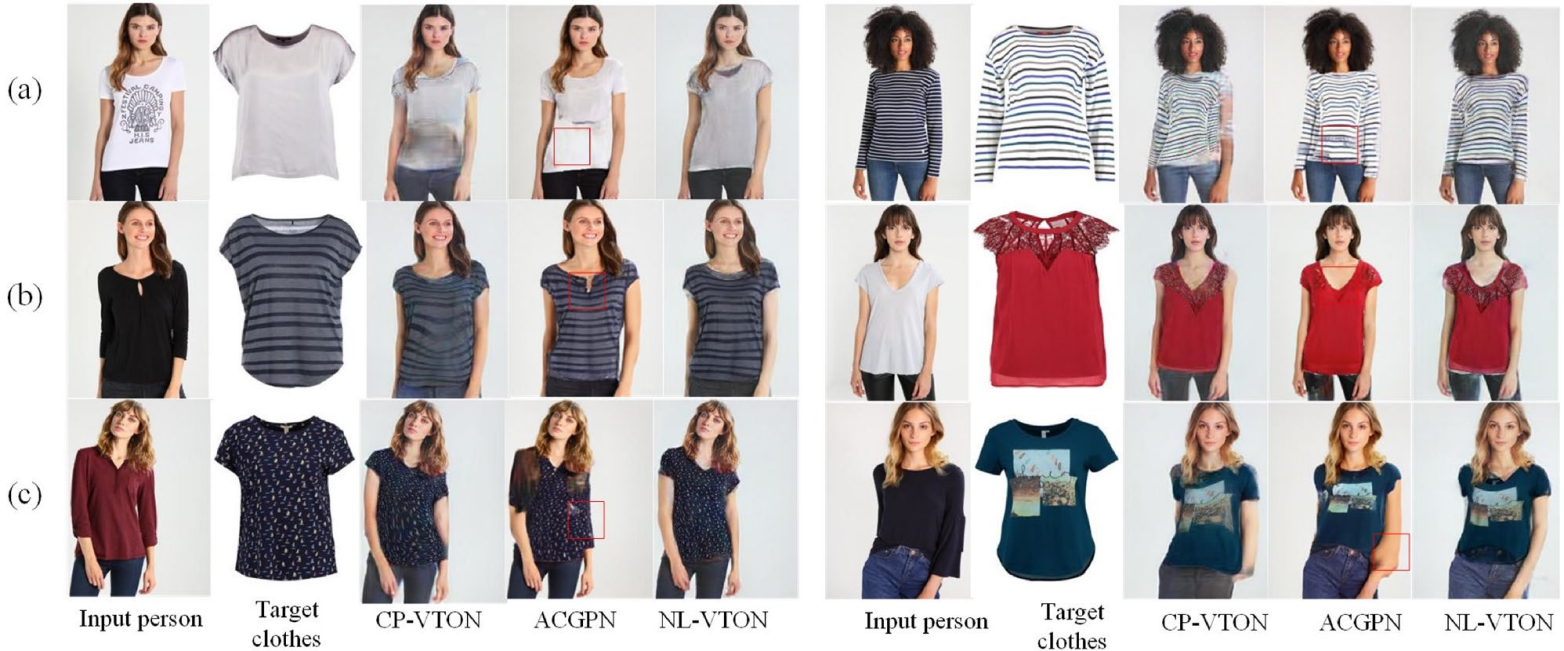
The more visual comparison with CP-VTON^1^, ACGPN^10^ and NL-VTON.

We visualize the virtual try-on results of NL-VTON, CP-VTON^1^, GarmentGAN^6^ and ACGPN^9^ in Fig. 9 (GarmentGAN does not provide source code, so the experimental results are limited to the experimental data provided in their paper). Compared with these methods, the proposed NL-VTON demonstrates the following three advantages:

1. Better feature preserving ability of bodys and clothes for complex target clothes. As shown in Fig. 8a, when the target clothes contains a lot of local patterns, the results of CP-VTON and ACGPN will lose some details, and the results of GarmentGAN has problems of blurring and local size enlargement. Comparatively speaking, the local patterns of “stay cool” generated by NL-VTON are clear, and their relative size and proportion are also reasonable.
2. Better feature preserving ability of bodys and clothes for complex human poses. As shown in Fig. 8b, when the person has a complex pose and there are some occlusion regions, the CP-VTON generates artifacts on occlusion regions. Although GarmentGAN avoids artifacts successfully, the clothes details become blurred. ACGPN generate result with clear arms, but the outline of the original image is retained on the sleeve part. NL-VTON also generate good result, but the arm is not as clear as ACGPN.
3. Better feature preserving ability of bodys and clothes for the case of big differences between the target clothes and the source clothes. As shown in Fig. 8c, when the clothes is transformed from a halter top to a short sleeve top, NL-VTON is able to preserve the features of the target clothes faithfully, while the results of CP-VTON and GarmentGAN have different degrees of blurring. Although ACGPN retains the details of the clothes, the original clothes collar is also retained

Figure 9 shows more visualization comparative results generated by CP-VTON^1^, ACGPN^10^ and NL-VTON. Obviously, the CP-VTON results are difficult to align the target clothes to body and generate unnatural distortions for complex details and striped patterns. ACGPN has the problem of retaining original clothes information, it can be seen from the line (a), the color of the original costume is retained in the result and in line (b), the collar of the original costume is retained; When the difference between the target clothes and the original clothes is too large, the boundary between the arm and the original clothes in the ACGPN result will be unclear, it can be seen from the line (c). While NL-VTON preserves the global shapes, texture structures and patterns details of the body and target clothes.

### Ablation studies

Ablation studies are conducted in this section. In the experiment, CP-VTON is chosen as baseline to demonstrate the performance of the proposed NLF-AM, the segmentation prediction network and the NL-GR loss.

#### Quantitative results

The comprehensive quantitative comparative results on SSIM and FID are shown in Table 5. Compared with the baseline CP-VTON^1^, the network b achieves 0.0162 and 7.461 improvements in SSIM and FID by employing the NL-GR loss, respectively; the network c achieves 0.0097 and 1.063 improvements in SSIM and FID by employing the NLF-AM, respectively; and the network d achieves 0.0462 and 8.511 improvements in SSIM and FID by employing both the non-local loss and the NLF-AM, respectively. It can be observed that: (1) Both the proposed NL-GR loss and NLF-AM have significant effects on improving the network; (2) The NL-GR loss has more obvious effects than the NLF-AM; (3) The improvements of adding both NL-GR loss and NLF-AM are better than the sum of the two single actions.

Further compared NL-VTON to other networks we can find that NL-VTON achieves the best results in both SSIM and FID by adding both the NL-GR loss, the NLF-AM and the human body segmentation prediction network.

The above results validity the proposed network and further demonstrate our idea that non-local operations and body segmentation maps are vital for the virtual try-on network.

**Table 5:** The quantitative evaluation of virtual try-on.

	Method	SSIM	FID
a	Baseline	0.7955	23.085
b	Baseline+$L_{grid}$	0.8117	15.624
c	Baseline + NLF-AM	0.8052	22.022
d	Baseline + NLF-AM + $L_{grid}$	0.8417	14.574
e	NL-VTON(Baseline + NLF-AM + $L_{grid}$ + Seg)	**0.8425**	**14.163**

#### Qualitative results

In this section, we compare the impacts of the proposed modules on the virtual try-on from a visual perspective.

**Figure 10:**
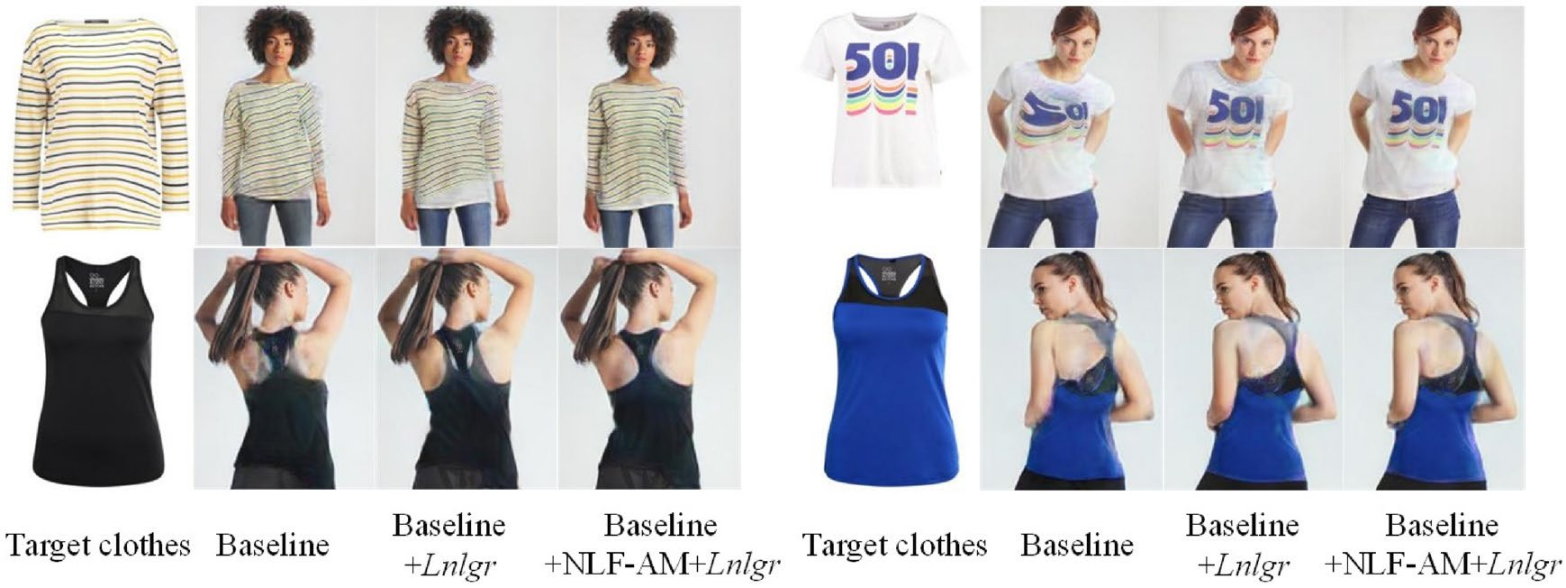
The visual comparison of the influence of non-local operations on virtual try-on results.

In order to match the quantitative experiment, this stage uses paired clothes for testing. As shown in Fig. 10, when adding the NL-GR loss to the baseline, the shapes of clothes become more complete, and the local patterns on clothes become clear. When further adding the NLF-AM to above network, the details of the pattern become clearer, the color becomes more realistic, and the local areas such as the collar and the cuff fit the body better.

**Figure 11:**
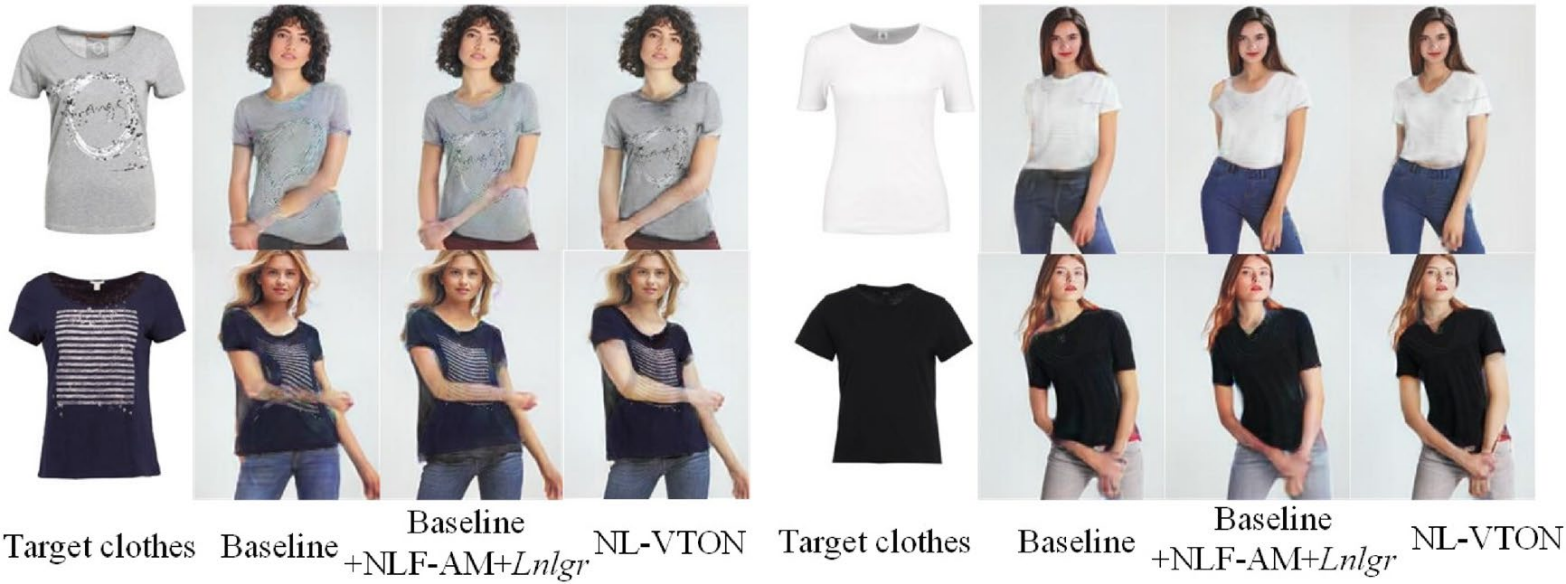
The visual comparison of the influence of human body segmentation prediction network on virtual try-on results.

In order to make the change of the arm more clear in the result, unpaired clothes are used for testing at this stage. Figure 11 shows the visualization results of further adding the human body segmentation prediction network (i.e, the complete NL-VTON) for handling complex poses and occlusion cases. It can be observed that although adding the NL-GR loss and the NLF-AM can solve the problem of blurriness on clothes details, the generated try-on results still suffer from artifacts on occlusion regions. When adding the human body segmentation prediction network, the results images show more complete human body shapes and clear arms, which demonstrate that the proposed segmentation prediction network can effectively predict different regions without compromising the retention of details.

**Figure 12:**
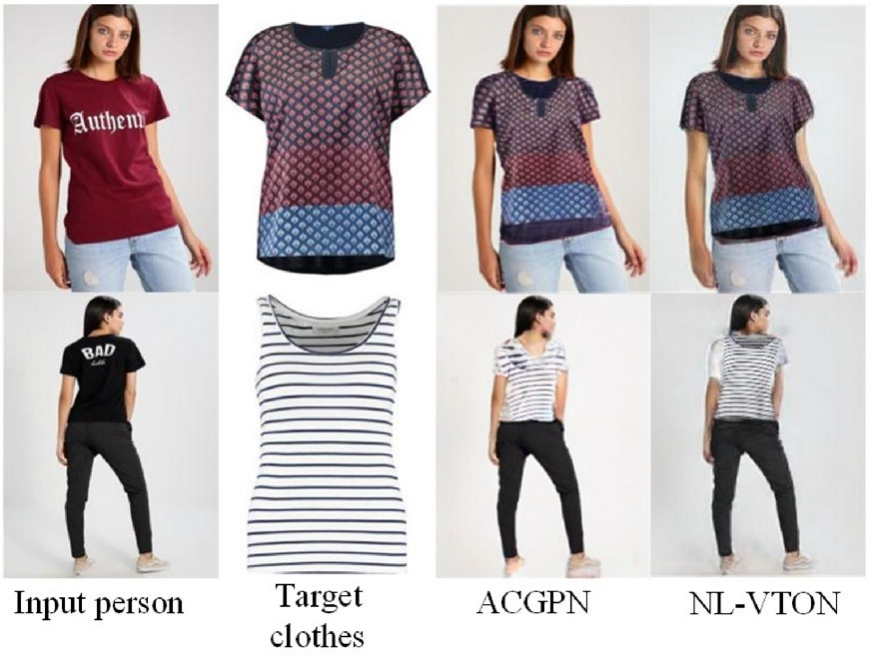
Some failure cases.

#### Limitations and discussion

The proposed NL-VTON has shown its evident advantage over the state-of-the-art methods. Nevertheless, there are several issues that need to be resolved for the 2D virtual-on task. For example, as Fig. 12 shown, both ACGPN and NL-VTON cannot distinguish the front and back of the collar very well and judge whether the person is forward or backward thus generate failed result. One possible solution is introducing dense human posture estimation and clothes key points into the 2D image based virtual try-on network. In addition, the existing quantitative evaluation can only test the results of wearing the same clothing. It needs to be combined with qualitative evaluation to judge whether the model is good or bad. Constructing a paired or ambiguous dataset for comprehensive testing will be the good solution.

## Conclusion

In this work, we propose a non-local virtual try-on network NL-VTON, to generate photo-realistic try-on results that preserve the features of both target clothes and human bodys. Our approach can alleviate blurriness on clothes details and artifacts on occlusion region of arms by integrating the proposed non-local FA module, the NL-GR loss and human body segmentation prediction network. The extensive visualized results demonstrate the feature preserving ability of NL-VTON and the quantitative comparison results also validity that the NL-VTON is superior to the state-of-the-art methods.

## Data Availability

Some or all data, models, or code generated or used during the study are available from the corresponding author by request.
