# Author Correction: NL-VTON: a non-local virtual try-on network with feature preserving of body and clothes

**DOI:** 10.1038/s41598-021-03035-y

**Published:** 2021-11-29

**Authors:** Ze Lin Tan, Jing Bai, Shao Min Zhang, Fei Wei Qin

**Affiliations:** 1 School of Computer Science and Engineering, North Minzu University, Yinchuan, 750021 China; 2 The Key Laboratory of Images & Graphics Intelligent Processing of State Ethnic Affairs Commission, Yinchuan, 750021 China; 3 School of Computer Science, Hangzhou Dianzi University, Hangzhou, 310018 China

Correction to: *Scientific Reports* 10.1038/s41598-021-99406-6, published online 07 October 2021

The Funding section in the original version of this Article was incomplete.

"Funding was provided by National Natural Science Foundation of China (Grant No. 61762003)."

now reads:

“This work was supported in part by National Natural Science Foundation of China under Grant 61762003, Grant 62162001 and Grant 61972121, in part by CAS "Light of West China" Program, and in part by Ningxia Excellent Talent Program.”

The original Article has been corrected.

